# Large Scale Screening and Quantitative Analysis of Site-Specific N-Glycopeptides from Human Serum in Early Alzheimer’s Disease Using LC-HCD-PRM-MS

**Published:** 2022-06-27

**Authors:** Lingyun Pan, Yu Lin, Jianhui Zhu, Jie Zhang, Zhijing Tan, David M. Lubman

**Affiliations:** 1 Department of Surgery, University of Michigan Medical Center, Ann Arbor, MI 48109, United States; 2 Experiment Center for Science & Technology, Shanghai University of Traditional Chinese Medicine, Shanghai 201203, China

**Keywords:** N-glycopeptides, PRM, LC-HCD-PRM-MS, Alzheimer’s disease, Biomarkers

## Abstract

Glycopeptide analysis by mass spectrometry may provide an important opportunity in discovery of biomarkers to aid in early detection of Alzheimer’s Disease (AD). In this work, we have used a NanoLC-Stepped-HCD-DDA-MS/MS platform and a NanoLC-Stepped-HCD-PRM-MS platform for large-scale screening and quantification of novel N-glycopeptide biomarkers for early detection of AD in patient serum. N-glycopeptides were retrieved from 10 μL of serum in patients with mild cognitive impairment (MCI, a prodromal phase of AD) and normal controls, respectively, after trypsin digestion, glycopeptide enrichment, fractionation, and NanoLC-Stepped-HCD-DDA-MS/MS or NanoLC-Stepped-HCD-PRM-MS analysis. Using a combination of Byonic, Byologic and Skyline softwares, we were able to accomplish both identification and label-free quantitation of site-specific N-glycopeptides between MCI and normal controls.

Differential quantitation analysis by Byologic showed that 29 N-glycopeptides derived from 16 glycoproteins were significantly changed in MCI compared to normal controls. Further, HCD-PRM-MS quantitative analysis of the selected N-glycopeptide candidates confirmed that EHEGAIYPDN138TTDFQR_HexNAc(4)Hex(5)-Fuc(2)NeuAc(1) from CERU, and VCQDCPLLAPLN156DTR_HexNAc(4)Hex(5)NeuAc(2) from AHSG can significantly discriminate MCI from normal controls. These two glycopeptides had the area under the receiver operating characteristic curve (AUC) of 0.850 (95% CI, 0.66–1.0) and 0.867 (95% CI, 0.68–1.0), respectively (p<0.05). The result demonstrates that changes in the expression level of the N-glycopeptides provide potential serum biomarkers for detection of AD at a very early stage.

## INTRODUCTION

Alzheimer’s disease (AD) is a neurodegenerative disorder that accounts for the majority of dementia cases [1–6], which affects around 30 million patients worldwide [7]. The neuropathological features of AD are amyloid deposits (plaques) of Aβ peptides derived from Amyloid β Precursor Protein (APP) and neurofibrillary tangles of tau protein [8–11]. Current evidence indicates that Aβ plaques begin to form many years before overt dementia, the gradual and progressive pathology which offers a potential target for early intervention [12]. Additionally, Mild Cognitive Impairment (MCI) has come to be recognized as an intermediate state of clinical impairment before advanced AD [9]. Due to changes in the brain triggered by AD before the presentation of initial symptoms, such as MCI, there is a need for early-stage diagnosis biomarker research [13]. To date, biomarkers in CSF have been extensively studied and the measurement of biomarkers in CSF has shown great diagnostic accuracy for AD. However, lumbar puncture is considered as a relatively invasive method in many countries [14]. In contrast to CSF, serum is easy to obtain with minimal discomfort to the patient. Serum can provide a rich source of biomarkers, as blood is in constant contact and exchange with all organs including the brain, where approximately 500 mL of CSF are absorbed daily into the circulating blood [14–20].

N-glycosylation of proteins is one of the most common posttranslational modifications [21]. In recent years, glycoproteomics has become an emerging field due to the discovery of unique glycopeptides associated with cancer [22], such as prostate [23], HCC [24,25], pancreatic and ovarian cancers [26,27]. It is believed that there are marked differences in levels of glycans involved in both protein O-GlcNAcylation and N-/O-glycosylation between patients and healthy individuals [9]. Also, recent glycomics studies of Cerebrospinal Fluid (CSF) or homogenates from brain showed altered glycosylation patterns in patients with MCI or AD, compared to normal controls [13,28,29]. Although limited by the low abundance and poor ionization efficiency, rapid development in sample preparation, enrichment strategies, and instrument functions have enabled glycopeptide analysis to complex glycopeptides and their quantitation [22,30,31].

Mass spectrometry has become a key technique for analysis of glycans as well as glycopeptides in the glycoproteomics research area due to instrumentation with high sensitivity, resolution, and mass accuracy [13,32]. In addition, different fragmentation methods have led to dissociation of ions at different peptide bonds, and therefore have different types of dominant fragment ions. Several MS dissociation methods have been reported to study intact glycopeptides such as Collision Induced Dissociation (CID), Higher-energy Collision Dissociation (HCD), Electron Capture/Transfer Dissociation (ECD/ETD) and Electron-Transfer Higher-Energy Collision Dissociation (EThcD) [22,32], where CID and HCD usually break glycosidic bonds and produce B and Y ions. The resulting MS3 spectrum of the Y1 ion facilitates database searching and *de novo* sequencing thus prompting the subsequent identification of the peptide backbone and associated glycosylation sites [33].

In the HCD spectra of glycopeptides, the attached peptide can be kept intact while bond breakages only occur at glycans. One can also use stepped HCD-MS/MS analysis of the complete glycopeptide structure by identification of glycan fragment ions and the b, y ions fragmented from the peptide backbones. As an alternative to non-targeted approaches, targeted quantitation strategies, such as Parallel Reaction Monitoring (PRM) use parent ion >daughter transitions to quantify glycopeptides and performs MS/MS scans on targeted precursors only, resulting in higher sensitivity and improved reproducibility compared to the precursor level quantification [34,35].

Herein, we have used a NanoLC-Stepped-HCD-DDA-MS/MS platform for screening of novel N-glycopeptide biomarkers for early detection of AD. We then established an LC-HCD-PRM-MS method which combines stepped HCD with PRM to quantitate the changes of targeted glycopeptides. With the application of the LC-HCD-PRM-MS method, we identified glycosylation changes in MCI compared with normal controls and quantified possible changes that might be potential new markers for AD in a limited patient cohort.

We chose 17 glycopeptide candidates from the identified glycopeptides for quantification analysis with PRM among individual patients. Our results confirmed that 6 N-glycopeptides were differentially expressed in MCI patients compared to normal controls, and two of these N-glycopeptides, EHEGAIYPDN138TTDFQR_HexNAc(4)Hex(5)Fuc(2) NeuAc(1) and VCQDCPLLAPLN156DTR_HexNAc(4)Hex(5) NeuAc(2), were increased. Further, the ROC curve analysis demonstrated that the AUC values of these two N-glycopeptides were 0.850 (95% CI, 0.66–1.0) and 0.867 (95% CI, 0.68–1.0), respectively.

## MATERIALS AND METHODS

### Materials

Reagents were purchased from Sigma (St. Louis, MO) unless otherwise specified. Human multiple affinity cartridges were purchased from Agilent (Santa Clara, CA). Sequencing-grade trypsin was purchased from Promega (Madison, WI). The 7K MWCO Zeba Spin Desalting columns were purchased from Thermo Scientific (Rockford, IL). The HILIC-packed TopTips were purchased from Glygen (Columbia, MD). The High pH Reversed-Phase Peptide Fractionation Kit was from Thermo Scientific (Waltham, MA). Pierce Albumin/IgG Removal Kit was purchased from Thermo Scientific (Rockford, IL). Amicon Ultra-0.5 Centrifugal Filter Devices (3,000 Molecular Weight Cut Off) were purchased from Millipore.

### Serum samples

Serum samples from patients with MCI (n=10) and healthy subjects (n=6) were provided courtesy of the Indiana Biobank, Indianapolis, IN, according to IRB approval and were collected under standard SOPs. Informed consent was obtained from all subjects. The controls are age matched with the MCI group, where the average age of the MCI patients is 73 years and that of the controls is 70. The conditions on the normal controls are such that the following conditions are required: 1. absolutely no cancer or other severe diseases, 2. No diabetes, 3. No neurological diseases, 4. No severe inflammation such as cirrhosis/fibrosis/pancreatitis, 5. No major head trauma-that is likely to impair cognition, 6. No alcoholism, 7. No Crohn’s disease, cystic fibrosis, or muscular dystrophy. The clinical features of patients are summarized in Table 1.

Samples were aliquoted and stored at −80°C until further use. Normal controls are particularly difficult to find in this age category because of the strictness of the conditions requested.

### Glycopeptides preparation for untargeted DDA-MS analysis

Depletion of serum high abundance proteins: 10 μL serum from 10 individual patients and 10 μL serum from 6 individual healthy subjects were mixed to constitute a pool, respectively, where the pooled samples from MCI and normal controls were each processed and analyzed. Because serum contains high abundance proteins (i.e., albumin, IgG, antitrypsin, IgA), the removal of high abundance proteins from serum was found to be necessary for proteomic analysis. 10 μL of this pooled serum sample was diluted by 200 μL PBS and filtered using a 0.22 μm filter. Then the filtered solution was loaded to the multiple affinity spin cartridge and centrifuged for 1.5 min at 100 g at RT where the flow-through fraction was collected. The cartridge was washed twice by 400 μL washing buffer (buffer A, Agilent Human 14 Multiple Affinity Removal System Spin Cartridges) by centrifugation at 2.5 min at 100 g at RT. and the washed fractions were collected and combined with the flow-through fraction, which was then dried down by freeze drying.

#### Trypsin digestion:

After removing the high abundance proteins using the multiple affinity cartridges, the glycopeptides were re-dissolved by NH4HCO3 (50 mM, pH 8.2). DTT (final concentration 50 mM) was added and incubated at 37°C for 30 min to reduce disulfide bonds, and 100 mM iodoacetamide (IAA) was added to alkylate the free thiol group for 30 min at RT. 50 mM DTT was added again to remove the extra IAA and then the solution was desalted by a ZebaTM Spin desalting column and dried down. The dried powder was re-dissolved by NH4HCO3 (50 mM, pH 8.2) and 3 μL trypsin (400 ng/μL) was added to the solution where the proteins were digested by trypsin (Ca. w/w=1:40) at 37°C overnight.

#### Glycopeptide enrichment by HILIC tips:

Glycopeptides were enriched using HILIC TopTips (Glygen, Columbia, MD) according to manufacturer’s instructions. Briefly, the HILIC TopTips were activated with water (20μL) 3 times, followed by equilibration with the binding buffer (85% acetonitrile containing 15 mM ammonium acetate, pH 3.5). The peptides were resuspended in binding buffer and loaded onto the HILIC TopTip, where the flow through was collected and reloaded 3 times to ensure complete binding. After glycopeptide capture, the HILIC TopTip was washed 3 times with 15 μL of 85% acetonitrile containing 15 mM ammonium acetate. The bound glycopeptides were then eluted with 15 μL of water 3 times and dried under vacuum.

#### Fractionation:

The glycopeptides were then fractionated using a High pH Reversed-Phase Peptide Fractionation Kit (Thermo Scientific) into eight fractions. The dried glycopeptides powder was dissolved in 300 μL 0.1% Trifluoroacetic Acid (TFA) solution. Briefly, the spin column was activated with acetonitrile (300μL) 3 times, followed by equilibration with 0.1% TFA. The glycopeptides were loaded to the column after column conditioning. The glycopeptides were washed by an 8-fraction washing buffer of which the percentage of ACN was increased from 5% to 50% and 0.1% Triethylamine(TEA) was decreased from 95% to 50%. After fractionation, fractions 1 and 2 were combined and fractions 6, 7 and 8 were combined, respectively, since these fractions have fewer peptides than other fractions based on the previous result. All fractions were dried down by vacuum.

LC-stepped-HCD-DDA-MS/MS: The enriched and fractionated glycopeptides (Frac1+2, Frac3, Frac4, Frac5, Frac6+7+8) from MCIs and normal controls were dissolved in 0.1% formic acid (FA) and analyzed with duplicate injections on an Orbitrap Fusion^™^ Lumos^™^ Tribrid^™^ Mass Spectrometer (Thermo) connected to a Dionex NanoLC system. A binary solvent system was composed of H2O containing 0.1% FA (A) and 80% CH3CN containing 0.1% FA (B) with a flow rate of 300 nL/min. Samples were separated on a 75 μm × 50 cm column (C18, 2μm, 100 Å; Acclaim PepmapTM RSLC, Thermo) under a 90 min linear gradient from 2 to 40% B. The MS instrument was operated in data dependent mode. The MS1 scans (m/z 400–1800) were acquired in the Orbitrap (120 k resolution, 4e5 AGC, 100 ms injection time) followed by Stepped HCD MS/MS acquisition of the precursors with the highest charge states in order of intensity and detection in the Orbitrap (60 k resolution, 2e5 AGC, 250 ms injection time). Stepped HCD was performed with optimized user-defined charge dependent reaction time supplemented by 31.5%, 35% and 38.5% HCD activation.

### Glycopeptides preparation for PRM-MS analysis

#### Depletion:

10 μL serum from 10 individual patients and 10 μL serum from 6 individual healthy subjects were thawed on ice, followed by Albumin/IgG depletion using Pierce Albumin/IgG Removal Kit, respectively, then the solution was desalted by a ZebaTM Spin desalting column and dried down.

#### Trypsin digestion:

The trypsin digestion of these 16 individual samples was consistent with that of the pooled samples

#### Glycopeptide enrichment by filter cut off:

Glycopeptides were enriched using 0.5 mL Centrifugal Filter Devices (3,000 Molecular Weight Cut Off) according to manufacturer’s instructions.

#### LC-PRM-MS identification and quantification of glycopeptides:

The enriched glycopeptides from MCI and normal controls were dissolved in 0.1% Formic Acid (FA) and subject to targeted quantitative analysis on the Orbitrap Lumos Mass Spectrometer (Thermo). The NanoLC system and the analysis column were the same as described above. The analytical gradient was 60 min long from 5 to 42% at a flow rate of 300 nL/min. Data acquisition was achieved in PRM mode which only performs MS/MS for the targeted precursors on the preloaded mass list. MS conditions were as described above with minor modifications on the MS1 scan range (700–1800 m/z) and the MS2 scan range (130–2000 m/z).

### Data analysis

All spectra were searched with Byonic (Protein Metrics), software for peptide/glycopeptide and protein identification based on MS tandem spectra, as described previously. A UniProt human protein database which includes 20 359 proteins was used for data searching by Proteome Discoverer 2.1 (Thermo Fisher Scientific, San Jose, CA) software using the Byonic (Protein Metrics) search engine with the following parameters (1) static modification, carbamidomethyl (C); (2) variable modifications, oxidation (M) and deamidation (N;Q) and N-glycan modifications (N); (3) up to 1 missed cleavage; (4) mass tolerance, 10 ppm for MS1 and 20 ppm for MS2; (5) fragment ion mass tolerance 0.01 Da. Results were filtered at a confidence threshold of Byonic score>150, Delta modification score>10, two-dimensional posterior error probability (PEP2D)<0.05, and two-dimensional local false discovery rate (FDR2D)<0.01. The manual check criteria included the retention time, the presence of oxonium ions, e.g. m/z 204.09 for HexNAc, 292.10 for NeuAc, 274.09 for NeuAc-H2O, 366.14 for HexHexNAc, 512.20 for HexHexNAcFuc, and 657.23 for HexNAcHexNeuAc.

Byologic (Protein Metrics Inc.) was used for automatic quantitative analysis, which uses MS1 raw data and Byonic search results as input. Byologic combines the list of identified glycopeptides, MS/MS spectra, XIC and precursor isotope plots in one window, so that complete information can be collected for quantitative analysis. With Byologic, the peak area of the XIC of a given glycopeptide was automatically integrated and normalized against the sum of peak areas of all glycopeptides in proteins identified in each MS run, providing a relative quantitation of each N-glycopeptide in the sample. The abundance of a site-specific glycoform was represented by the sum of the glycopeptides bearing the same glycan at the glycosite, where the peptide sequences may be different in the case of miscleavage or deamidation.

Skyline software was used for the quantification of selected glycopeptides using the Y1 ion (peptide+HexNAc), HexNAc oxonium ions (m/z 204.09) and/or Y2 (peptide+2HexNAc), Y3 (peptide+2HexNAc-Hex), b/y ions for identification. The setting of Skyline software analysis includes peptide and transition settings to generate a spectral library for quantitative glycopeptides. Also, the Skyline analysis workflow has been shown in a previous study [36]. A spectral library was then generated, where oxonium ions, Y1 ions and the optimal b, y ions were selected to identify the glycopeptides. After completing the required settings, the Skyline was then ready for importing the raw data of individual samples for quantitative analysis. The integral peak areas of the Y1 ions were automatically calculated and exported from Skyline, which were used for glycopeptide quantification and data normalization. The relative abundance of each glycopeptide was calculated by normalizing its peak area to the sum of peak areas of all targeted glycopeptides in each sample. Principal component analysis (PCA), a multivariate statistical analysis, was also used to aid in the visualization of differences between the cohorts.

### Statistical analysis

The comparison of intact N-Glycopeptides identified by different Byonic scores in two groups use GraphPad Prism 7 (La Jolla, CA). Statistical analysis was performed using SPSS IBM version 26.0. In this study, all the data were manually inspected, strict mass tolerances (10 ppm/0.01 Da precursor/product ions) were applied while the assignments of glycopeptides with Byonic scores>150 were confirmed with MS/MS spectra. To control FDR2D well below 1%, the cut off of the Byonic score was set as 150.

## RESULTS

### Glycopeptides preparation for untargeted DDA-MS analysis

**Figure 1:**
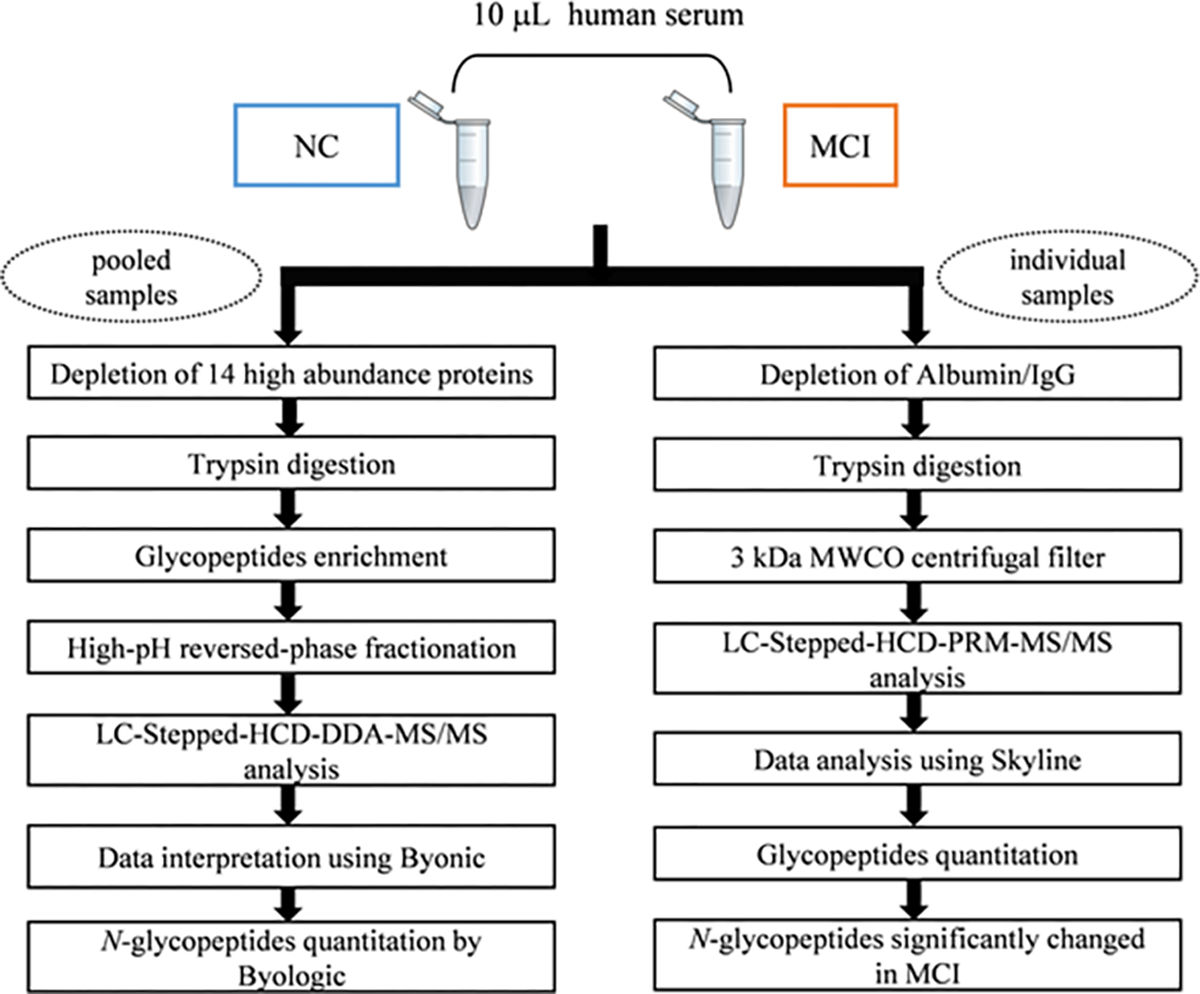
Workflow of profiling site-specific N-glycopeptide changes between MCI and normal groups.

The workflow used in this work is outlined in Figure 1. 10 μL serum from 10 individual patients and 10 μL serum from 6 individual healthy subjects were mixed to constitute a pool, respectively. Then the pooled serum sample was depleted to remove the 14 high abundance proteins by the multiple affinity spin cartridge. After removing the high abundance proteins, the glycopeptides were digested with trypsin, enriched using HILIC TopTips, and then fractionated using a High pH Reversed-Phase Peptide Fractionation Kit (Thermo Scientific) into eight fractions, and finally followed by NanoLC-HCD-MS/MS analysis in duplicate on the Orbitrap Fusion Lumos Tribrid Mass Spectrometer (Thermo). All spectra were searched with Byonic (Protein Metrics). Intact N-glycopeptides at each glycosylation site were quantitated using Byologic (Protein Metrics). Finally, statistical analysis was performed to evaluate the differentially expressed site-specific glycopeptides in MCI compared to normal controls (Figure 1).

It is notable that although most of high abundance serum proteins have been removed by depletion cartridge, there was still a small amount of high abundance proteins left in the depleted sample. To ensure consistency, these remainder glycopeptides in the pooled samples from MCI or normal controls were not deleted before normalization. The method reproducibility of the workflow was evaluated in a previous study in our group [25]. Briefly, the method reproducibility was evaluated by three independent experiments of fractions from a random serum sample, and the result indicates a good reproducibility of the workflow.

#### Glycopeptides score distribution:

All the N-glycopeptides were identified by Byonic software. The Byonic score, utilizes stringent criteria that includes the presence of glycan-specific oxonium ions and assignable b/y peptide peaks for confident glycopeptide matching. In this study, intact N-Glycopeptides identified by different Byonic scores were compared in normal controls and MCI samples with FDR2D<0.01, PEP2D<0.05. As shown in Figure S1, whether in normal controls or MCI groups, the number of glycopeptides below Byonic score 150 is the smallest, and the highest is in a Byonic score range between 300 and 450. In Control groups, the number of N-glycopeptides below 150 Byonic score accounted for only 5% of the total, while in the MCI group it was about 6%. Hence, in subsequent studies, a minimum Byonic score of 150 is considered as a highly confident assignment of N-glycopeptides to ensure the reliability and can be used to compare the differences between two groups, thus ensuring the reliability of the Glyco-map and that low abundance glycopeptides were not considered. Figure S2 shows the percentage of glycan-specific peptides, that is, the percent of sialylated or fucosylated glycopeptides identified by different Byonic scores in Control and MCI groups. Fucosylated and/or sialylated glycopeptides accounted for most of the N-glycopeptides in either the normal controls or the MCI groups.

#### Identification of intact N-glycopeptides:

To verify differentially expressed glycopeptides for biomarker candidates with early AD diagnostic potential, we compared the glycopeptides from MCI and normal controls. Analysis of the pooled serum samples yielded 666 glycopeptides corresponding to 81 glycoproteins and 144 glycosites.

**Figure 2:**
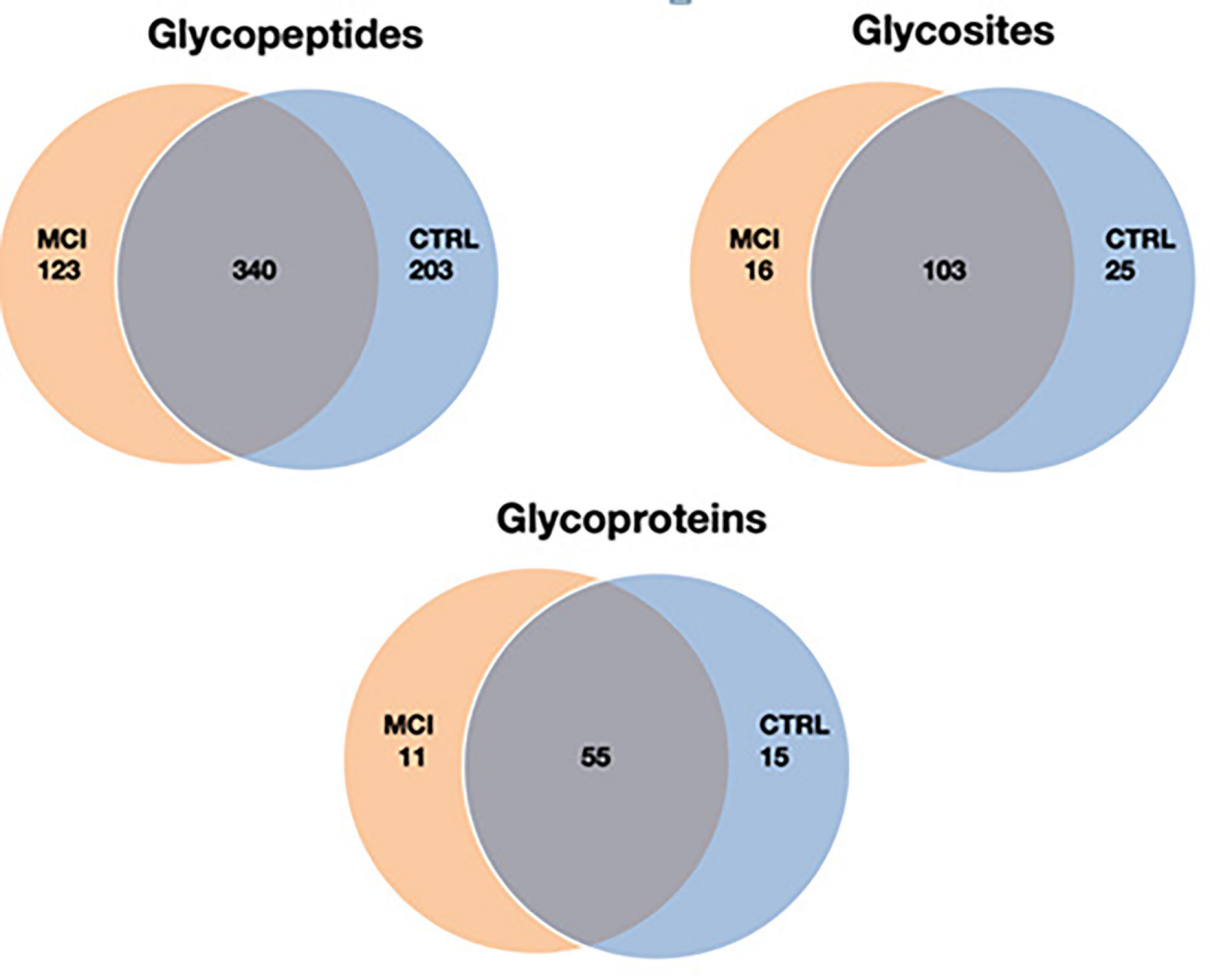
Venn diagram comparison of identified glycopeptides, glycosites, and glycoproteins between MCIs and normal controls.

Figure 2 depicts, for example, 463 known glycopeptides in MCIs and 543 known glycopeptides in normal controls, corresponding to 66 and 70 glycoproteins, 119 and 128 glycosites in MCIs and normal controls, respectively.

**Figure 3:**
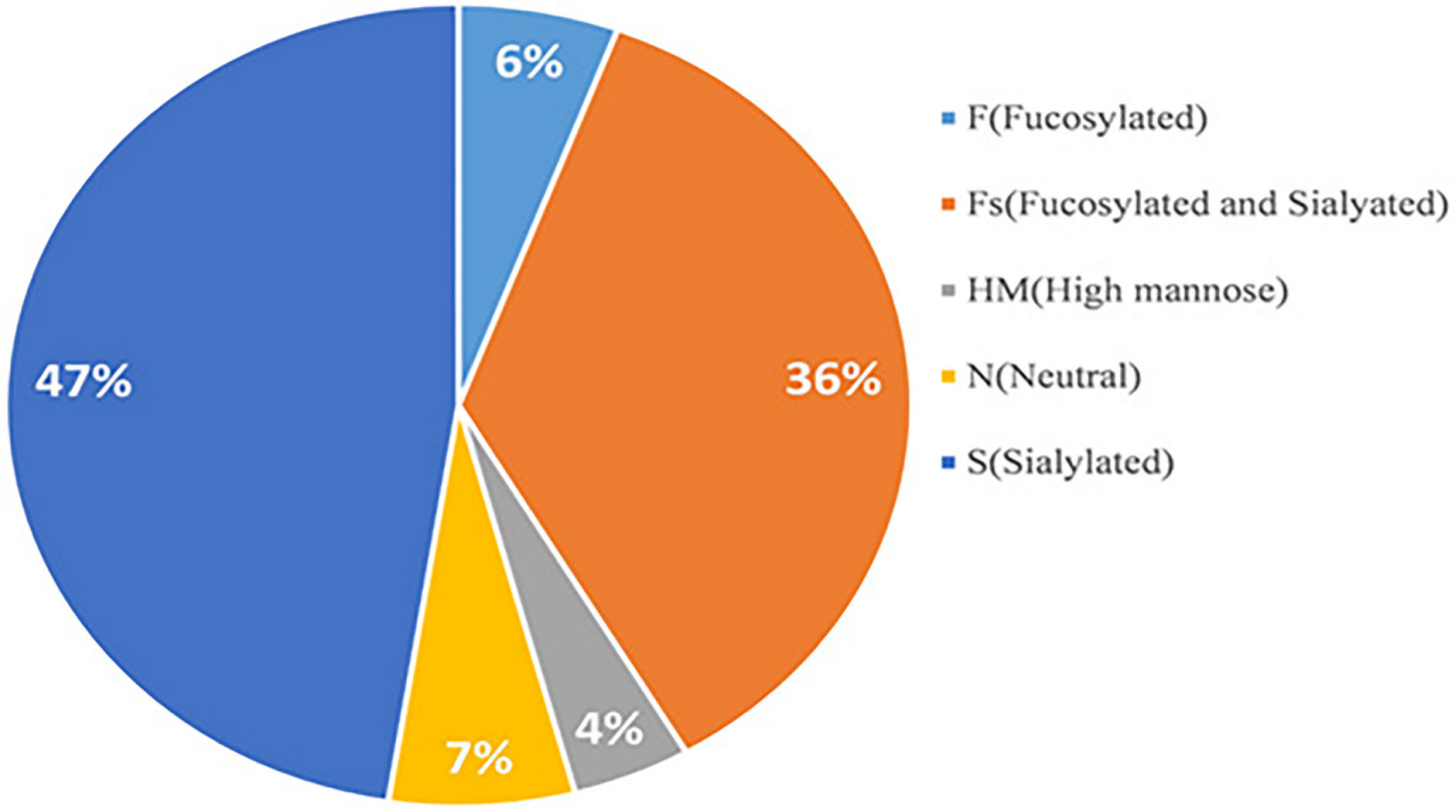
Glycosylation types of serum glycoproteins identified in the pooled MCI and normal samples. Note: (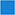) F(Fucosylated); (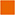) Fs(Fucosylated and Sialyated); (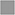) HM(High mannose); (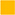) N(Neutral); (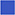) S(Sialylated).

Among the glycopeptides, approximately 6% were only fucosylated, 44% were only sialylated and 39% were both fucosylated and sialylated (Figure 3). A small portion of glycopeptides contained high-mannose and neutral N-glycans. All the N-glycopeptides spectral matches are listed in Table S1, including the glycosite, peptide sequence and glycan composition. Moreover, the number of glycosylation sites and unique glycopeptides of each glycoprotein are summarized in Table S2. The N-glycopeptides identified in each fraction of the pooled MCI or normal samples are shown in Tables S3 and S4 respectively. To simplify the annotation of N-glycan composition, a 4-digit nomenclature was used in the order of HexNAc(a)_Hex(b)_Fuc(c)_NeuAc(d) (HexNAc=N-acetylhexosamine; Hex=Hexose; Fuc=Fucose; NeuAc=sialic acid; a, b, c, and d, the number of the monosaccharides).

#### LC-stepped-HCD-DDA-MS/MS of N-glycopeptides:

The N-glycopeptides and their relative glycosites ratio (MCI/CTRL) when the relative abundance of one of two groups is expressed more than 0.5 are summarized in Table 2.

As shown in Table 2, 29 N-glycopeptides from 16 glycoproteins were significantly changed (Ratio>2 or Ratio<0.5). Among these differentially expressed N-glycopeptides, 12 up-regulated and 17 down-regulated N-glycopeptides were changed in MCI compared to normal controls. Furthermore, there were a total of 10 different glycan compositions in these 29 N-glycopeptides. Most of the glycan compositions were HexNAc(4)Hex(5) Fuc(2)NeuAc(1) and HexNAc(4)Hex(5)NeuAc(2). Some of these glycoproteins have been reported to serve as candidate biomarkers for AD and MCI. These include: Attractin [37,38], Ceruloplasmin [39,40], Prothrombin [41], Antithrombin-III [42], Alpha-1-antichymotrypsin [43], Leucine-rich alpha-2-glycoprotein [44], Alpha-2-HS-glycoprotein [45,46], Heparin cofactor 2 [38], Afamin [16,47], N-acetylmuramoyl-L-alanine amidase [48]. The role of some detected glycoproteins in MCIs such as Heparin cofactor 2 are not explained by known neuropathologies or the literature where there is no indication of it being involved in the prevention and treatment of cognitive decline and dementia.

Representative MS/MS spectra of fingerprint N-glycopeptides of VCQDCPLLAPLN156DTR with glycan HexNAc(4)Hex(5) Fuc(2)NeuAc(1) is shown in Figure S3. Oxonium ions such as HexNAc (m/z 204.09), HexHexNAc (m/z 366.13), NeuAc (m/z 292.10), and HexNAcHexNeuAc (m/z 657.23) were present at high intensity in the spectra. B and y fragments from the peptide backbone were observed as well but at low intensity. Glycosidic fragments with the loss of monosaccharides from the parent glycopeptide were well characterized in the Stepped HCD-MS/MS spectra. Specific diagnostic fragment ions further provided detailed structural information such as core and outer arm fucosylation (marked by red arrows). For example, the diagnostic fragment ion of HexHexNAcFuc (m/z 512.20) confirmed the presence of the outer-arm fucose residue, while the ions of peptide+HexNAc+Fuc (m/z 1060.99) confirmed the core fucose residue.

### Glycopeptides preparation for targeted PRM-MS analysis

The experimental workflow for top 2 proteins depletion for PRM-MS is also shown in Figure 1. In this work we used individual samples rather than pooled samples, and then used an Albumin/IgG removal kit to deplete individual samples as being simpler compared to the use of the top 14 high abundance proteins removal kit.Next we chose 17 glycopeptides from 10 proteins which were found in most samples for PRM analysis (Table S5).

10 patients and 6 individual healthy subjects were used in this study. The PCA analysis of normalized results of MS/MS fragments showed two clusters of these two groups, indicating these two groups can be distinguished to a certain extent through the information of these 17 glycopeptides.

#### LC-HCD-PRM-MS of N-glycopeptides:

From statistical analysis of the relative abundance of each glycopeptide quantified using the Skyline software, we found that 6 glycopeptides from 5 glycoproteins were significantly changed.

Table S6 summarized the targeted glycopeptides for PRM quantitation, where their corresponding integral peak areas, precursor ions and product ions are listed. The scatter plots of these differentially expressed glycopeptides between MCI and normal controls are shown in Figure S4.

Of these 6 glycopeptides that change significantly, EHEGAIYPDN138TTDFQR_HexNAc(4)Hex(5)Fuc(2)NeuAc(1) from CERU and VCQDCPLLAPLN156DTR_HexNAc(4)Hex(5) NeuAc(2) from AHSG were increased when comparing MCI and normal controls (Table 3).

**Figure 4:**
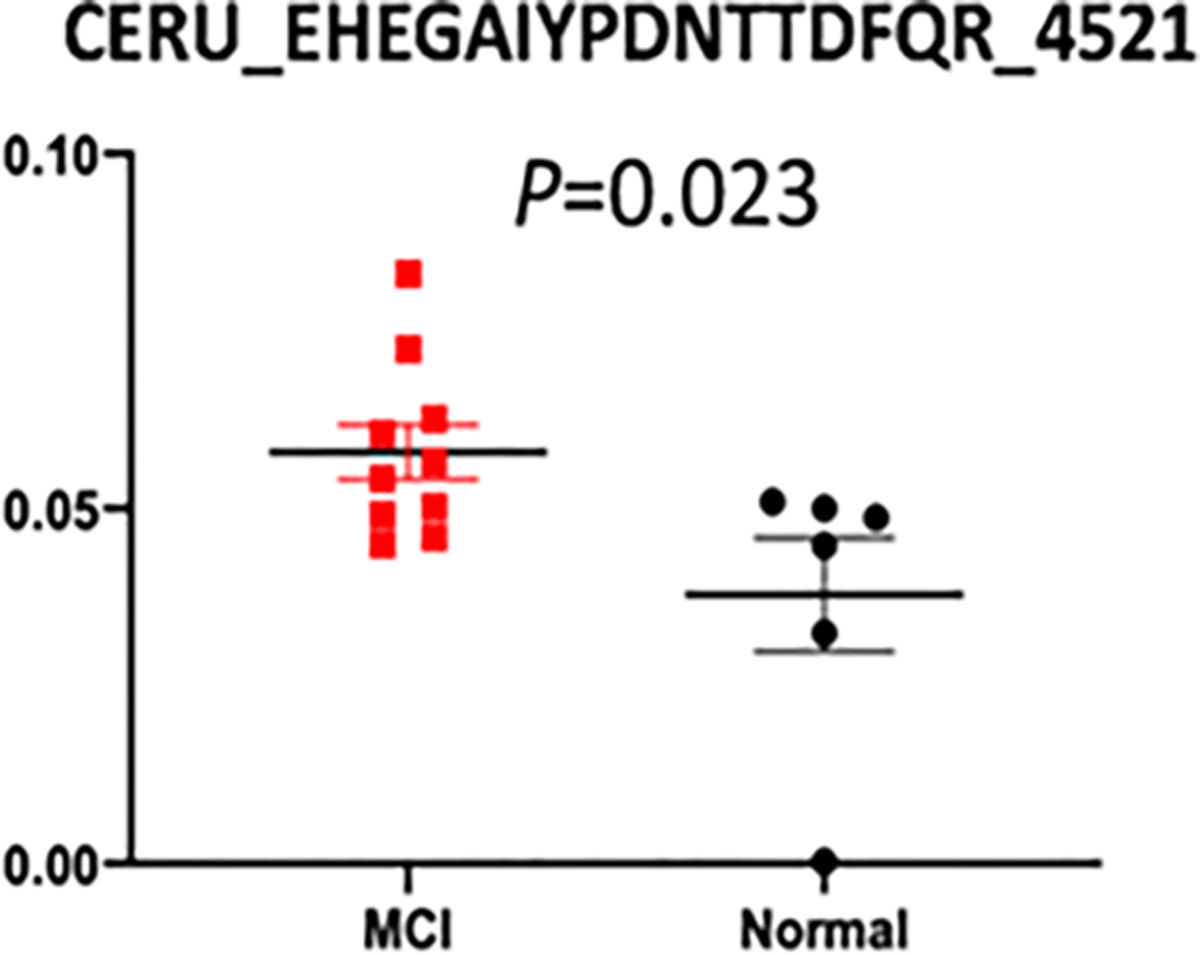
Scatter plot of relative abundance of glycopeptide EHEGAIYPDN138TTDFQR_ HexNAc (4) Hex (5) Fuc (2) NeuAc (1) in MCI patients (red squares) and normal controls (black circles), respectively. The P-value was marked below the figure.

**Figure 5:**
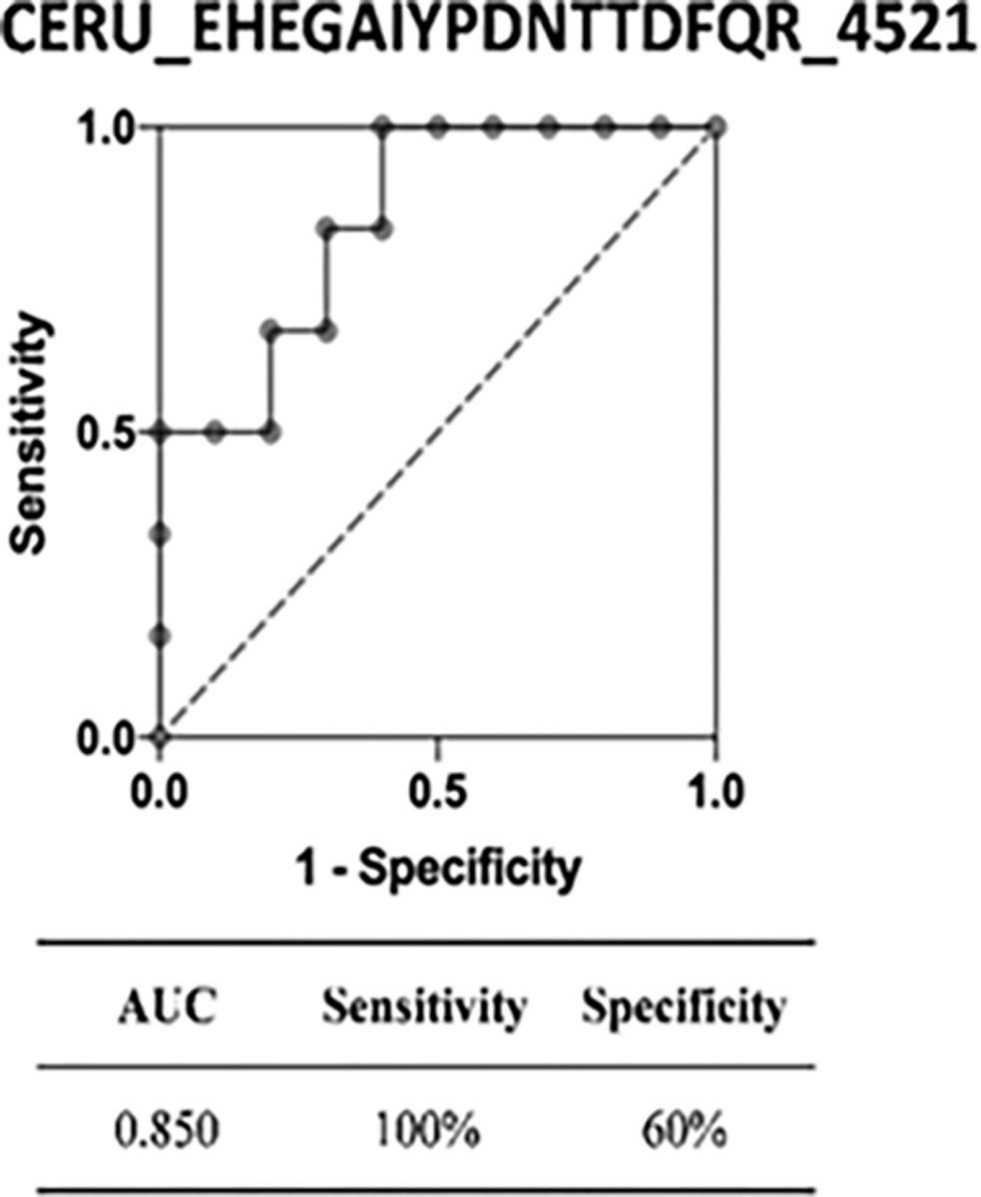
ROC analysis of glycopeptide EHEGAIYPDN138TTDFQR_ HexNAc (4) Hex (5) Fuc (2) NeuAc (1) between MCI patients vs. normal controls.

**Figure 6:**
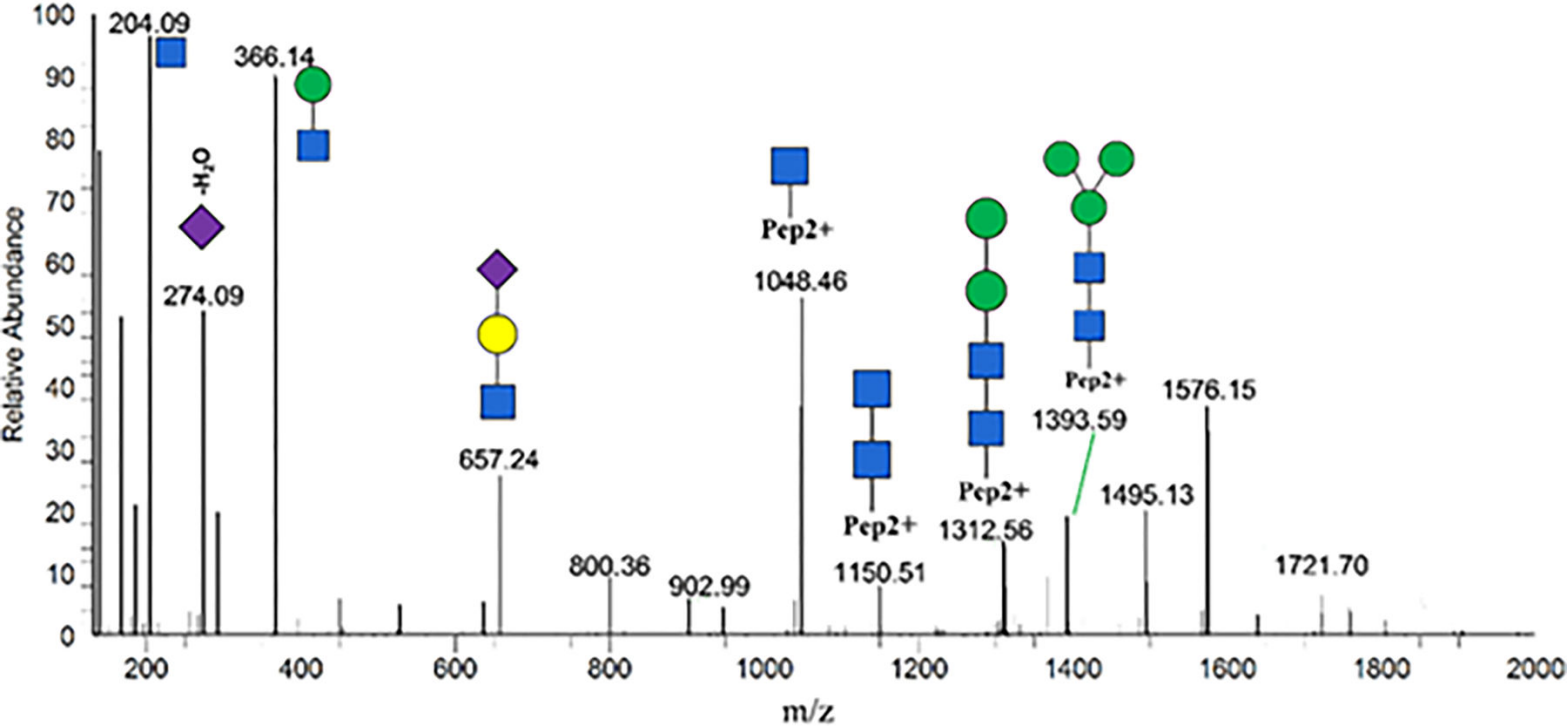
A representative tandem mass spectrum of glycopeptide EHEGAIYPDN138TTDFQR_HexNAc(4)Hex(5)Fuc(2) NeuAc(1). The symbols used in the structural formulas: Blue square: HexNAc; Green circle: Man; Yellow circle: Gal; Purple diamond: NeuAc.

**Figure 7:**
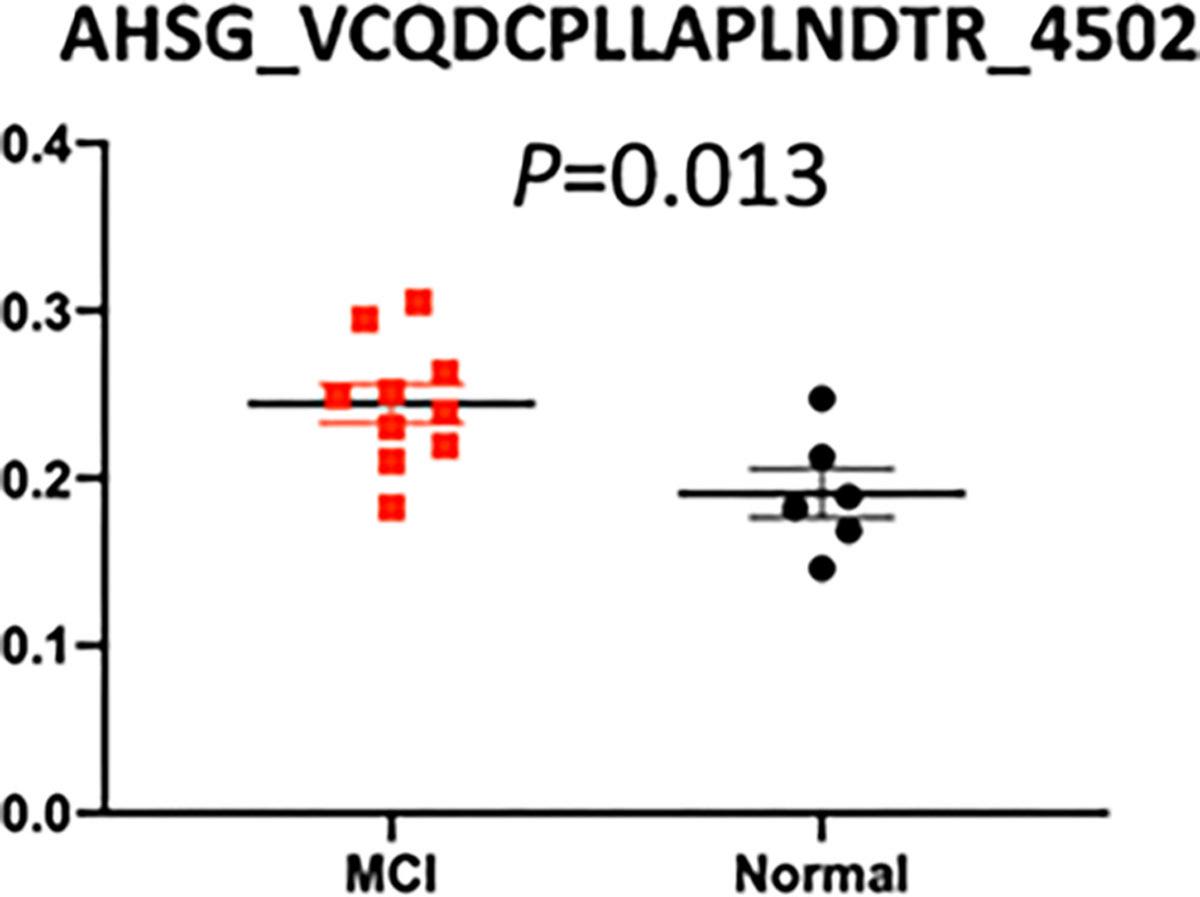
Scatter plot of relative abundance of glycopeptide VCQDCPLLAPLN156DTR_ HexNAc (4) Hex (5) NeuAc (2) in MCI patients (red squares) and normal controls (black circles), respectively. The value was marked below the figure.

**Figure 8:**
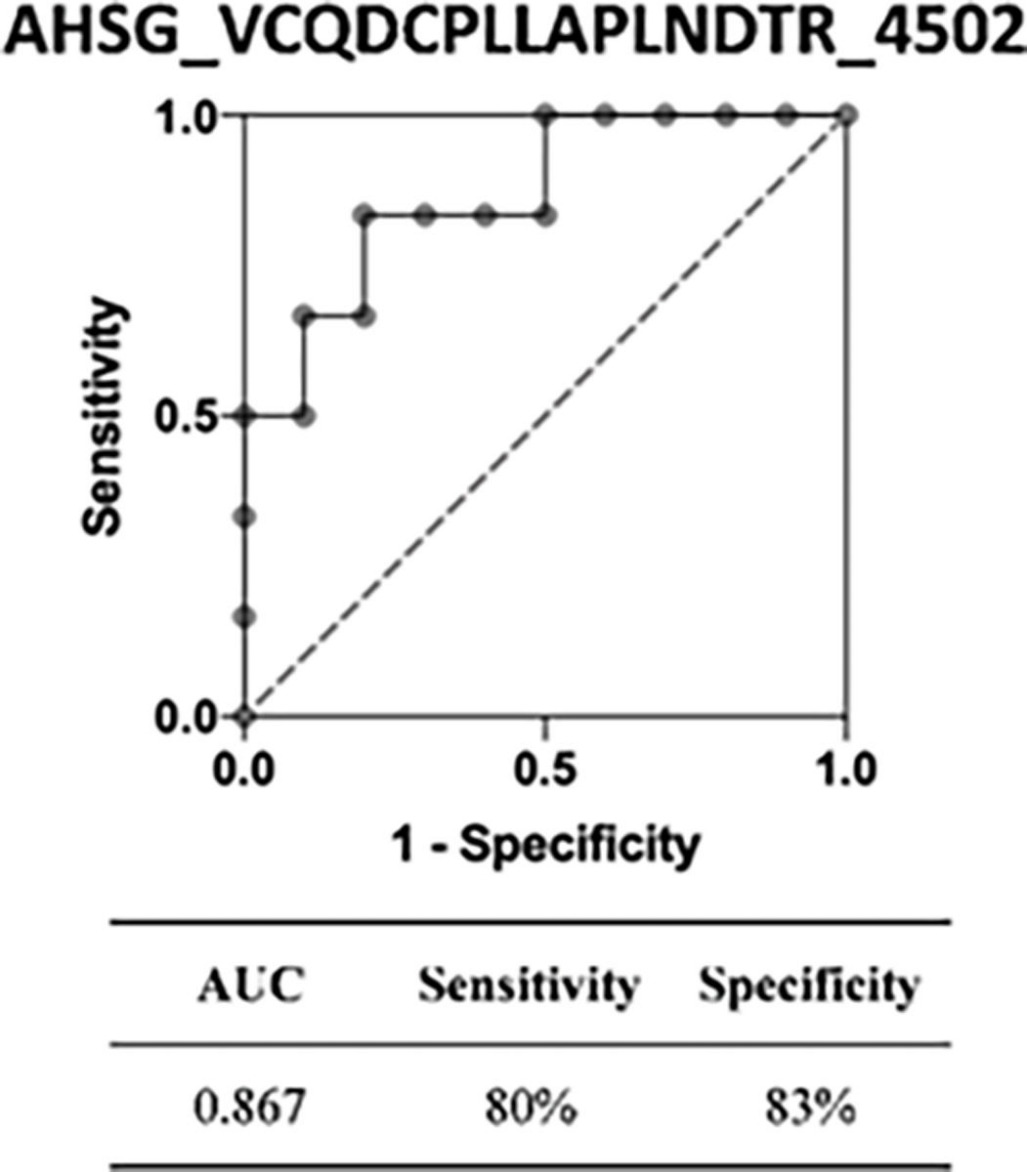
ROC analysis of glycopeptide VCQDCPLLAPLN156DTR_ HexNAc (4) Hex (5) NeuAc (2) between MCI patients vs. normal controls.

**Figure 9:**
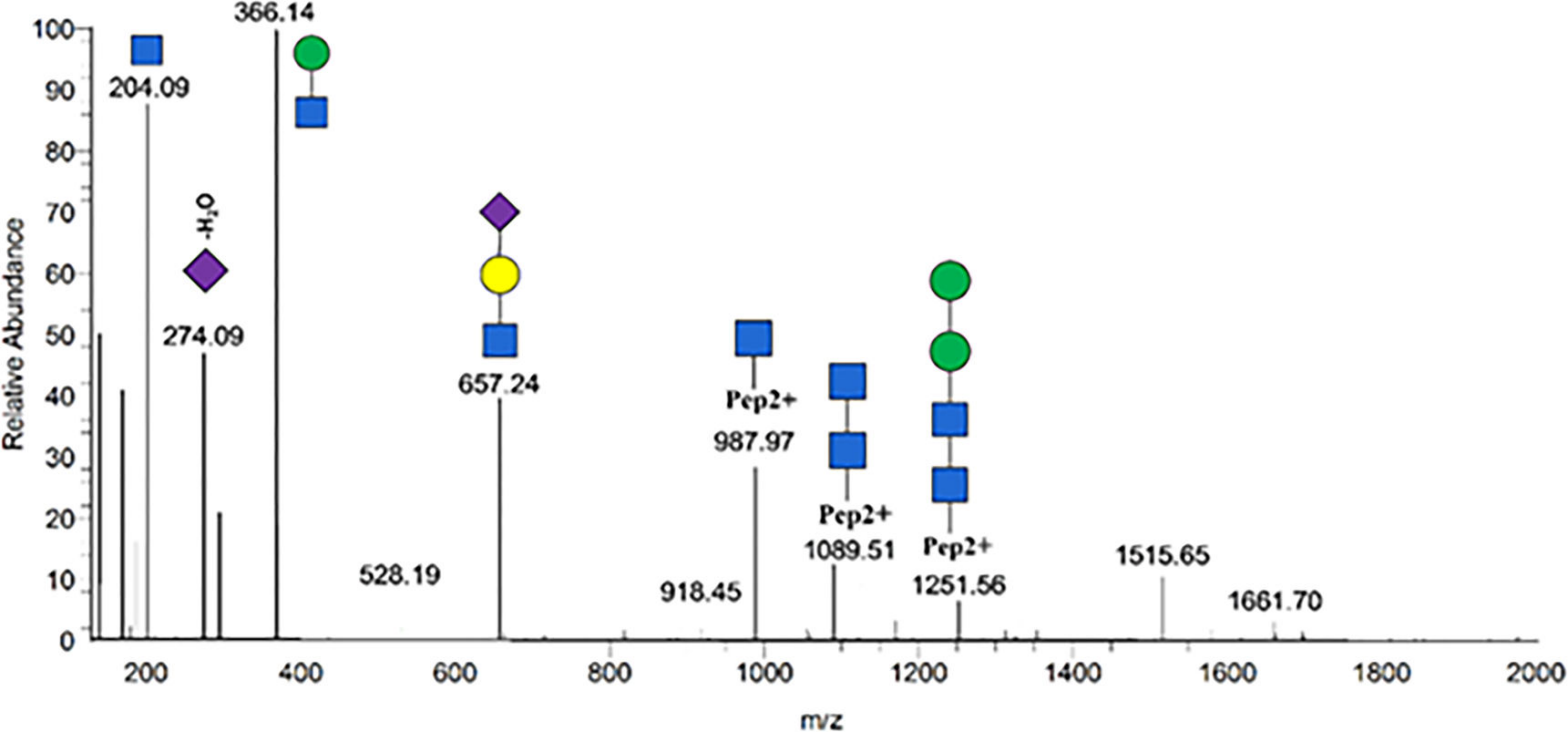
A representative tandem mass spectrum of glycopeptide VCQDCPLLAPLN156DTR_HexNAc (4) Hex (5) NeuAc (2). The symbols used in the structural formulas: Blue square: HexNAc; Green circle: Man; Yellow circle: Gal; Purple diamond: NeuAc.

The receiver operating characteristic curve (ROC) analysis of these two glycopeptides are shown in the figures, respectively, where the area under ROC curve (AUC) of EHEGAIYPDN138TTDFQR_HexNAc(4)Hex(5)Fuc(2)NeuAc(1) is 0.850 (95% CI, 0.66–1.0) with a sensitivity of 100%, a specificity of 60% and the area under ROC curve (AUC) of VCQDCPLLAPLN156DTR_HexNAc(4) Hex(5)NeuAc(2) is 0.867 (95% CI, 0.68–1.0) with a sensitivity of 80%, a specificity of 83%. The tandem mass spectra of these two glycopeptides are shown in Figures 4–9.

#### PCA analysis:

**Figure 10:**
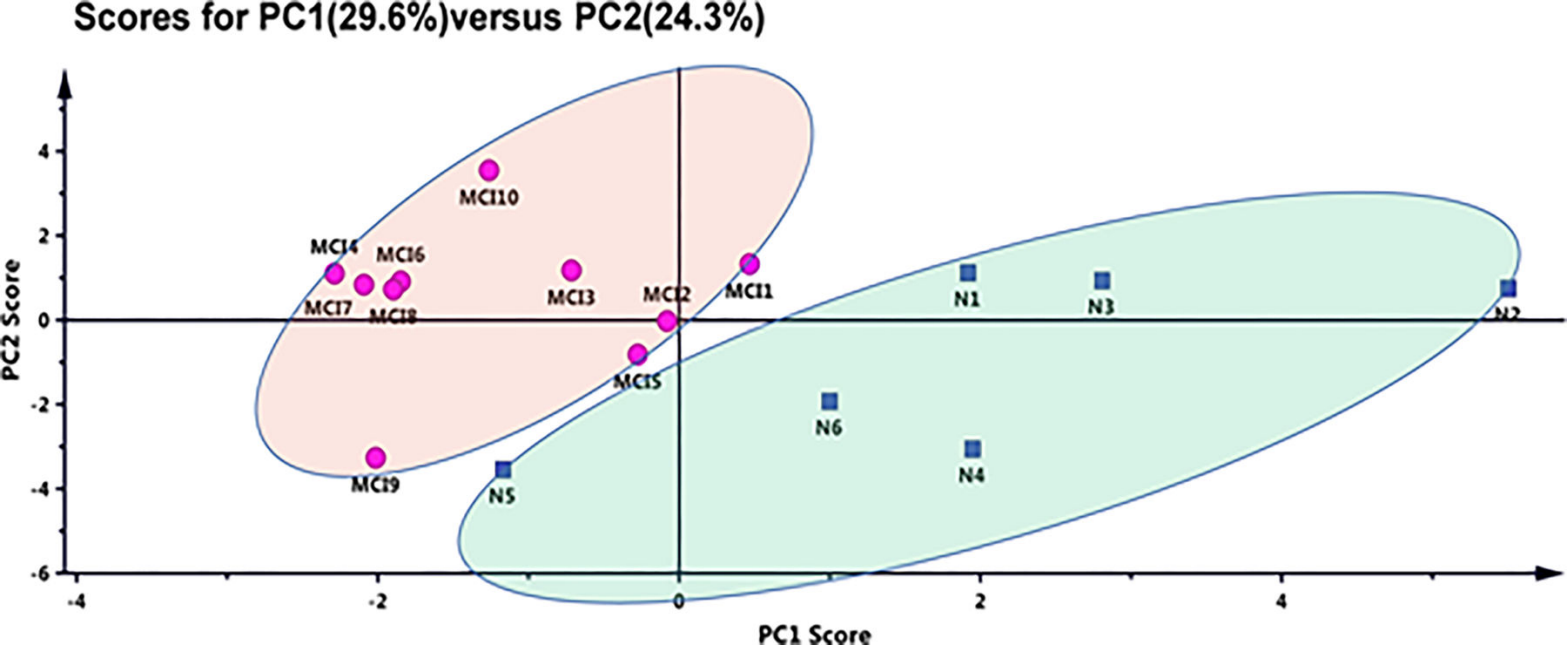
Principal component analysis of MCI versus normal controls. Pink circle: MCI; Blue square: Normal controls.

PCA is a commonly used chemometric tool to analyze the differences between two sets of data with multiple variables. By the unsupervised PCA method, we observed discrete trends between the MCI and normal controls and judged whether there were outliers. Figure 10 exhibits PCA plots of MCI versus normal controls. Interestingly, only through the normalized PRM data of these 17 peptides, distinct differences between the MCI and normal controls were clearly observed. The PCA plots indicate that these 17 peptides are representative (Figure 10).

## DISCUSSION

There is general agreement that a major problem with the almost uniformly disappointing clinical trials conducted to date for potential AD therapeutics is that they employed patients with moderate to advanced disease. The present preliminary results suggest that the changes in the expression levels of the N-glycopeptides could serve as potential serum biomarkers for early AD diagnosis and could provide insight into the molecular mechanisms involved in the pathogenesis of AD.

Gizaw et al. [49] reported that the expression levels of the serum and CSF protein N-glycans such as the total expression of bisecting and multiply branched glycoforms were significantly increased in AD patients. Through the in-depth study using differential quantitation analysis, we found that most of the glycoforms that changed significantly in MCI groups were bisecting N-glycans.

Pooled samples were used to identify potential markers to decrease the excess of time and cost required to run the individual samples, thus ensuring the reliability of the Glyco-map and low abundance glycopeptides were not used. In our study, a confidence threshold of Byonic score>150 was set to ensure the reliability of the glycopeptides and exclude low abundance glycopeptides.

Once the serum samples have been obtained, they are generally processed to remove high-abundance proteins or aliquoted into more convenient sizes [50]. In this study, we found that the Top 2 High Abundance Depletion Method is sufficient for depletion when performing PRM of targeted glycopeptides. Based on the Top 2 protein depletion method, we used the Albumin/IgG Removal Kit instead of the Human 14 Multiple Affinity Removal System Spin Cartridge for depletion, and then used a 3 kDa filter, which can not only achieve the effect of enriching glycopeptides, but also desalting and performing buffer exchange. In addition, the sample processing time is greatly reduced due to the reduction of the steps in the depletion.

A proteomic study by Johnson et al. [17] on a large-scale analysis of AD’s brain tissue and CSF revealed that astrocyte/microglial metabolism module had a strong relation with AD, and it was significantly enriched in AD genetic risk factors. By comparing with the top 100 proteins by module eigenprotein correlation value in this module in AD brain, we found that 3 proteins yielded by pooled serum samples including complement C4A(C4A), complement C4B (C4B), and clusterin (CLU) were consistent with the proteins in this module. Among them, the N-glycopeptide signals of the first two proteins were very low, while a glycopeptide of CLU with a relatively strong signal was significantly changed, where the N-glycopeptide was LAN374LTQGEDQYYLR with glycan HexNAc(4)Hex(5) Fuc(2)NeuAc(1). Thus, LANLTQGEDQYYLR was added to Table S5 as a candidate.

Interestingly, the glycopeptides VTPDNFSTIIK attached at Asn270 glycosite, LCDNLSTK attached at Asn288 glycosite, VNSTELFHVDR attached at Asn421 glycosite and CIAFIVDNFSK attached at Asn37 glycosite (Table S2) were identified for the first time in our experiment, which may benefit from the advanced Stepped HCD-MS/MS fragmentation technique.

EHEGAIYPDN138TTDFQR with glycan HexNAc(4)Hex(5)Fuc(2) NeuAc(1) is a Ceruloplasmin-derived peptide, which was increased in MCI (P<0.05) compared with controls. A similar observation was reported that relatively high levels of Ceruloplasmin(CP) were associated with accelerated disease progression in people with MCI and underlying Aβ-pathology [51], and also that the high level of CSF ceruloplasmin is significantly related to HIV-associated neurocognitive disorders [52]. However, the role of CP in Alzheimer’s disease (AD) is unclear. CP, which is the major copper binding protein of plasma, plays an important role in intersecting oxidative stress and neuroinflammation, since it is an acute phase protein whose expression is induced during inflammation [51]. Surprisingly, CP can reduce cellular iron burden [52], which has been reported to be elevated in the AD cortex [53]. It has been reported that CP if present in increased concentration could aggravate the damaging effect of pro-inflammatory stimuli in brain by modulating microglial activation [54]. Therefore, higher ceruloplasmin may increase neuroinflammation. Kristinsson et al. found AD patients have serum CP concentrations that are similar to healthy individuals [55]. In our study, CP has shown significant differences at the glycopeptide level, so that it may have value as a biomarker.

VCQDCPLLAPLN156DTR with HexNAc(4)Hex(5)NeuAc(2) is an Alpha-2-HS-glycoprotein-derived peptide, which was increased in MCI (P<0.05) compared with controls. Alpha-2-HS-glycoprotein (or Fetuin-A; AHSG) is part of the cystatin superfamily of cysteine protease inhibitors, which encompasses a series of closely related proteins that are synthesized mostly in the liver [56]. Fetuin-A is a multi-functional protein, including as an important inhibitor of ectopic calcification acting on the systemic level [57,58], one of the steps that can be active in disease progression in axial SpA patients [59] and a TGFb1 antagonist in advanced cancers and fibrosis [60]. In AD patients, fetuin-A is considered pro-inflammatory [61]. Some studies have also reported that in the plasma and CSF of AD patients, they found that compared with the control group, the concentration of fetuin-A was decreased with a significant increase in the concentration of TNF-α [45,62]. Inconsistent with the results at protein levels, we found that at the peptide level, peptides with significant differences were elevated in patients with MCI.

## CONCLUSION

In this study, we designed an integrated protocol of several sample preparation techniques combined with stepped-HCD-DDA-MS/MS to perform quantitative profiling of the N-glycan structures of glycoproteins in serum between disease states. Changes in expression level of N-glycopeptides in serum of MCI patients versus normal controls can be monitored quantitatively by this protocol. More specifically, 29 N-glycopeptides from 16 glycoproteins were changed significantly, most of which are biantennary glycans with fucose and/or sialic acid residues.17 glycopeptide targets were further selected for PRM quantification using a LC-HCD-PRM-MS method. 6 N-glycopeptides showed significantly different changes in MCI compared to normal controls, and two of these N-glycopeptides, EHEGAIYPDN138TTDFQR_HexNAc(4)Hex(5) Fuc(2)NeuAc(1) and VCQDCPLLAPLN156DTR_HexNAc(4)Hex(5)NeuAc(2), were increased. Further, the ROC curve analysis demonstrated the AUC values of these two N-glycopeptides were 0.850 (95% CI, 0.66–1.0) and 0.867 (95% CI, 0.68–1.0), respectively. These two biomarker candidates may represent disease-specific blood signatures of early AD.

This work demonstrated a workflow for studying the quantification of novel N-glycopeptide biomarkers for early detection of AD in patient serum using the NanoLC-Stepped-HCD-DDA-MS/MS platform and LC-Stepped-HCD-PRM-MS platform. The automated relative quantitative analysis using Byologic software, with the NanoLC-Stepped-HCD-MS/MS platform, greatly reduced the range of biomarker candidates. In addition, quantitative analysis using skyline software, with the LC-Stepped-HCD-PRM-MS platform, further confirmed the differentially expressed glycopeptides. These markers still need to be validated in a larger sample set but appear to be promising candidates for early detection of AD.

## Supplementary Material

supp info

STable1

STable2

STable3

STable4

STable5

STable6

## Figures and Tables

**Table 1:** Characteristics of study participants.

Characteristic	Control	MCI	Total
No.	6	10	16
Gender (M/F)	4/2	08/2	12/4
Age (mean ± SD)	70.5 ± 11.4	73.5 ± 8.3	72.4 ± 9.3
Diabetes status, No. (%)	0(0%)	3(30%)	3(19%)

**Table 2:** N-Glycopeptides significantly differentially expressed in MCI as compared to CTRL (Ratio >2.0-fold or Ratio <0.5-fold) when the relative abundance of one of two groups expressed more than 0.5.

Uniprot	Protein	Glycopeptide	Glycosite	Ratio
				(MCI/CTRL)
O75882	Attractin	VFHIHNESWVLLTPK_2500^a^	N428	2.08
		VFHIHNESWVLLTPK_3501	N428	2.79
		VFHIHNESWVLLTPK_3600	N428	3.33
P00450	Ceruloplasmin	EHEGAIYPDNTTDFQR_4521	N138	N.D.
P00734	Prothrombin	YPHKPEINSTTHPGADLQENFCR_4502	N143	0.24
P01008	Antithrombin-III	LGACNDTLQQLMEVFK_4502	N128	0
		WVSNK_4521	N224	0.43
P01011	Alpha-1-antichymotrypsin	KLINDYVKNGTR_4502	N186	0.38
		KLINDYVKNGTR_5613	N186	0
		KLINDYVKNGTR_5603	N186	0
		LINDYVKNGTR_4502	N186	0.24
P01042	Kininogen-1	HGIQYFNNNTQHSSLFMLNEVK_4502	N169	0
		ITYSIVQTNCS_5613	N205	0.4
P02749	Beta-2-glycoprotein 1	VYKPSAGNNSLYR_4521	N162	4.28
P02750	Leucine-rich alpha-2-glycoprotein	KLPPGLLANFTLLR_4521	N186	0
		LPPGLLANFTLLR_4502	N186	0.09
		MFSQNDTR_4521	N325	9.69
P02765	Alpha-2-HS-glycoprotein	VCQDCPLLAPLNDTR_4502	N156	2.19
		VCQDCPLLAPLNDTR_4521	N156	4.29
P02790	Hemopexin	CSDGWSFDATTLDDNGTMLFFK_4502	N64	0
		NGTGHGNSTHHGPEYMR_4521	N246	3.75
		NGTGHGNSTHHGPEYMR_4501	N246	2.39
		SWPAVGNCSSALR_5613	N187	0.4
P04070	Vitamin K-dependent protein C	RNRTFVLNFIK_4502	N355	N.D.
P05155	Plasma protease C1 inhibitor	DTFVNASR_4512	N238	0.4
P05156	Complement factor I	LISNCSK_4521	N494	0.06
P05546	Heparin cofactor 2	DFVNASSK_4521	N188	13.55
P43652	Afamin	YAEDKFNETTEK_4521	N402	0.35
Q96PD5	N-acetylmuramoyl-L-alanine amidase	LEPVHLQLQCMSQEQLAQVAANATK_4502	N367	0

Note:

a The glycanforms were presented as: HexNAc(a)Hex(b)Fuc(c)NeuAc(d); a, b, c and d are the number of each monosaccharide. For example, in the first line of the table, the glycan_2500 stands for HexNAc (2) Hex (5).

**Table 3:** List of precursor from Ceruloplasmin and Alpha-2-HS-glycoprotein for PRM quantification.

Glycoprotein	Glycopeptide	Glycana	Charge	Precursor ion (m/z)	Product ion(m/z)
Ceruloplasmin	ELHHLQEQN[+2350.83035]VSNAFLDK	4512	4	1093.964	1113.045, 1214.585, 1295.611
					, 1376.638
	EHEGAIYPDN[+2205.79284]TTDFQR	4521	3	1366.883	1048.464, 1150.003, 1231.030
					, 1312.056
Alpha-2-HS-glycoprotein	VC[+57.02146]QDC[+57.02146] PLLAPLN[+2204.77244]DTR	4502	3	994.91	1052.014, 1153.554, 1234.580
					, 1315.606
	VC[+57.02146]QDC[+57.02146] PLLAPLN[+2205.79284]DTR	4521	3	1326.551	1052.014, 1153.554, 1234.580
					, 1315.606
	VC[+57.02146]QDC[+57.02146] PLLAPLN[+2221.78776]DTR	4611	3	1331.883	1052.014, 1153.554, 1234.580
					, 1315.606

**Note:** aThe glycan forms were presented as: HexNAc(a)Hex(b)Fuc(c)NeuAc(d); a, b, c and d are the number of each monosaccharide. For example, in the first line of the table, the glycan_4512 stands for HexNAc (4)Hex (5) Fuc(1) NeuAc (2).

## ABBREVIATIONS

AD: Alzheimer’s Disease
APP: Amyloid β Precursor Protein
AUC: Area Under the Curve
BEVs: Brain-secreted Extracellular Vesicles
CID: Collision Induced Dissociation
CSF: Cerebrospinal Fluid
DTT: Di-Thio-Threitol
ECD/ETD: Electron Capture/Transfer Dissociation
EThcD: Electron-Transfer Higher-Energy Collision Dissociation
HCD: Higher-Energy Collisional Dissociation
HILIC: Hydrophilic Interaction Liquid Chromatography
IAA: Iodoacetamide
LC: Liquid Chromatography
MCI: Mild Cognitive Impairment
MS: Mass Spectrometry
MWCO: Molecular Weight Cut-Off
PCA: Principal Component Analysis
ROC: Receiver Operating Characteristic
PRM: Parallel Reaction Monitoring
RT: Room Temperature
SOP: Standard Operating Procedure
EIC: Extracted Ion Chromatogram
