# Supplementary material for: Large Scale Screening and Quantitative Analysis of Site-Specific N-Glycopeptides from Human Serum in Early Alzheimer’s Disease Using LC-HCD-PRM-MS
Source: J Proteomics Bioinform. Author manuscript; Available in PMC 2023 Jun 23. (PMC10289803)
Supplement: supp info [file NIHMS1855164-supplement-supp_info.docx]

Large Scale Screening and Quantitative Analysis of Site-Specific *N*-Glycopeptides from Human Serum in Early Alzheimer’s Disease Using LC-HCD-PRM-MS

Lingyun Pan^1,2‡^, Yu Lin^1‡^, Jianhui Zhu^1*^, Jie Zhang^1^, Zhijing Tan^1^, and David M. Lubman^1*^

^1^ Department of Surgery, University of Michigan Medical Center, Ann Arbor, MI 48109, United States

^2^ Experiment Center for Science & Technology, Shanghai University of Traditional Chinese Medicine, Shanghai 201203, China

**Supporting Information**


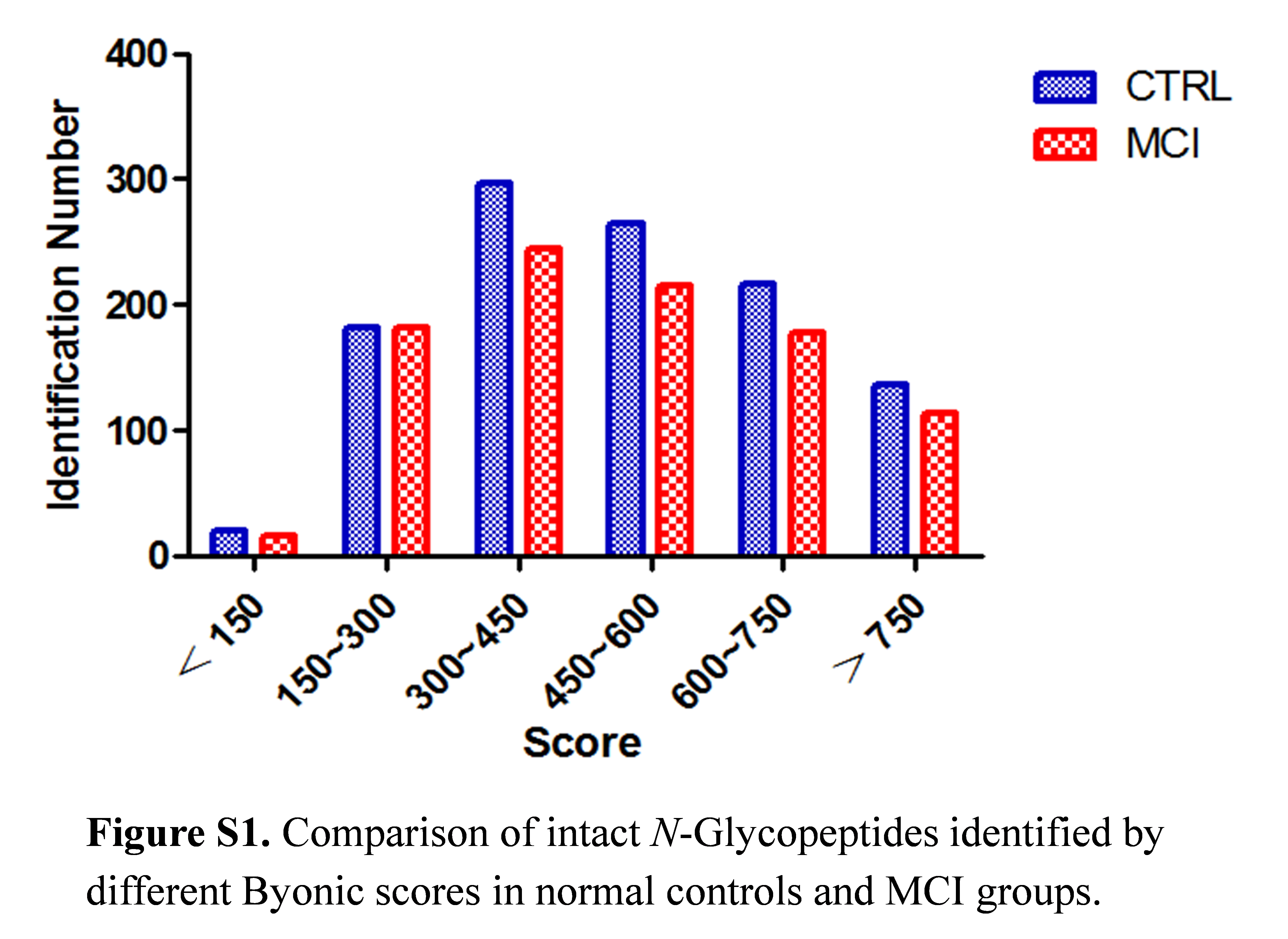


**Figure S1.** Comparison of intact *N*-glycopeptides identified with different Byonic scores in normal controls vs. MCI group.


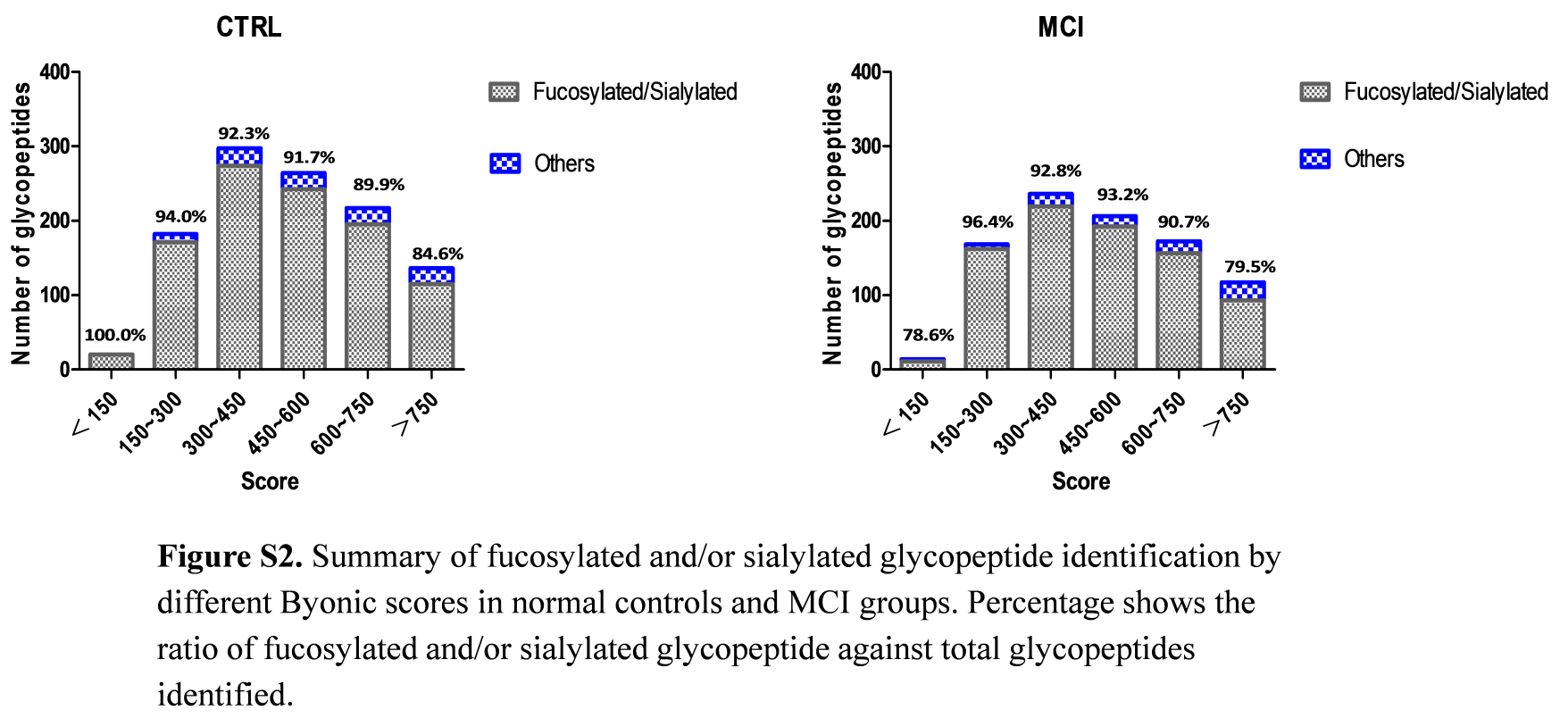


**Figure S2.** Summary of fucosylated and/or sialylated glycopeptide identification with different Byonic scores in normal controls and MCI group, respectively. Percentage represents the ratio of fucosylated and/or sialylated glycopeptides against the total glycopeptides identified.


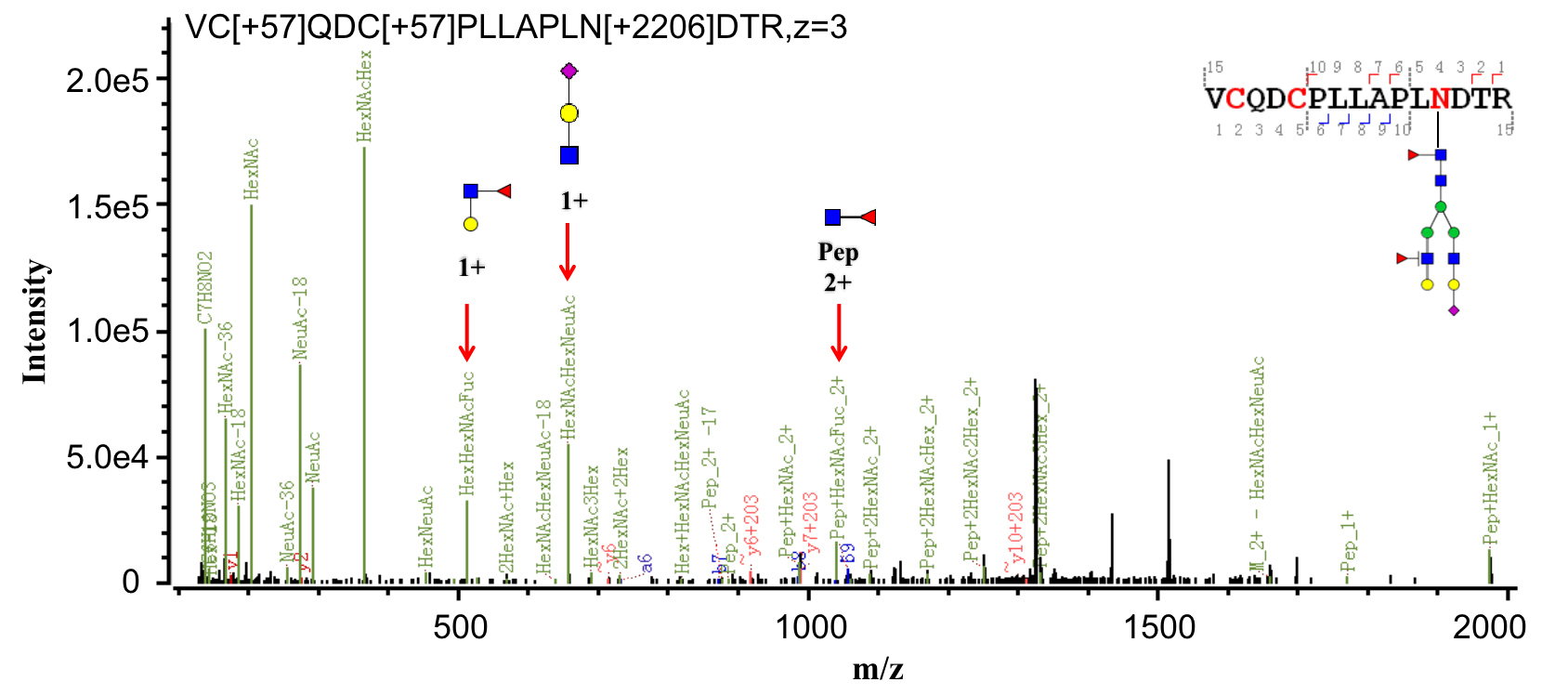


**Figure S3.** MS/MS spectrum of the glycopeptide VCQDCPLLAPLNDTR with 4HexNAc-5Hex-2Fuc-1NeuAc attached at site Asn156.


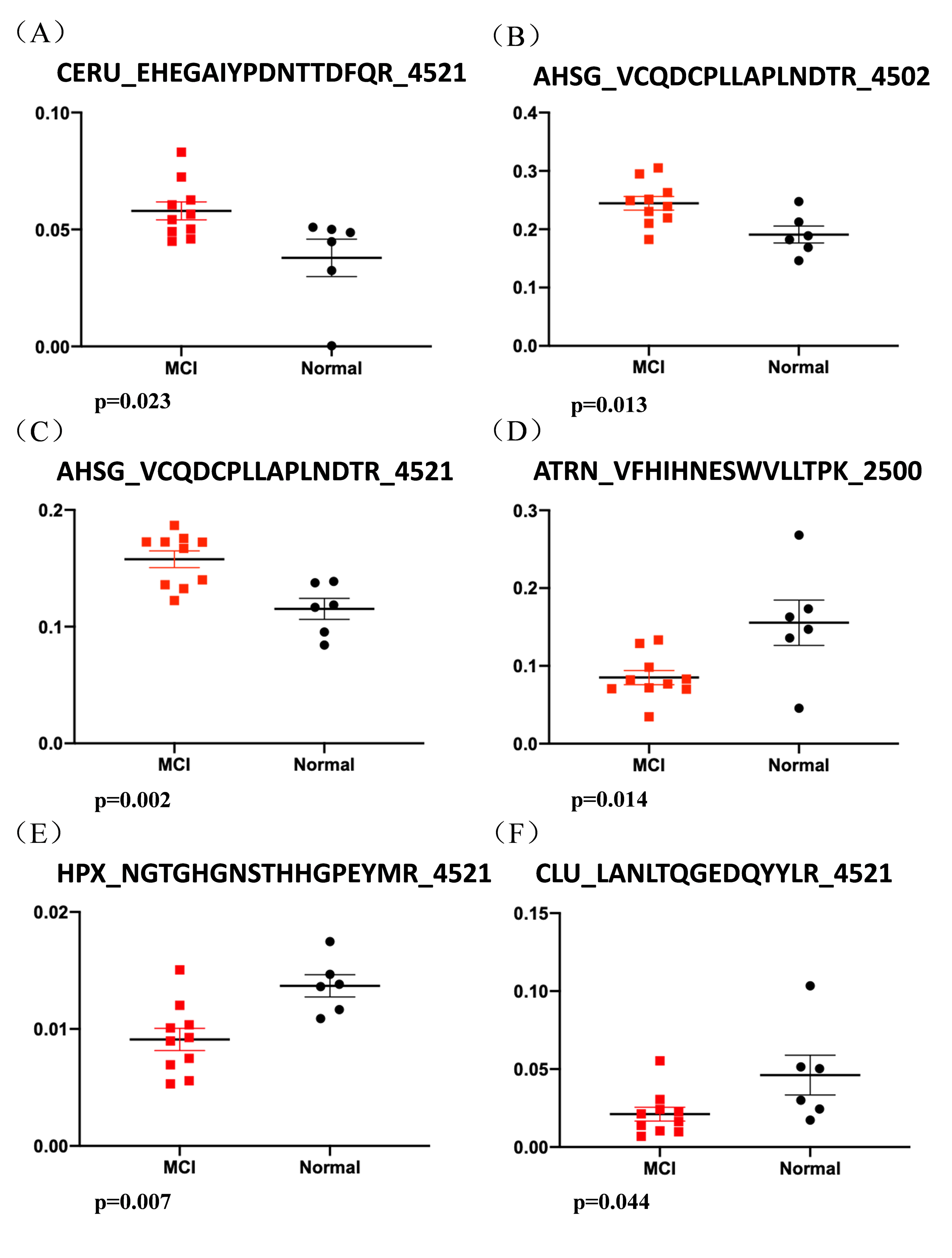


**Figure S4.** Scatter plots of the six differentially expressed glycopeptides between MCI patients vs. normal controls. A t-test comparison was made between MCI group and normal group. Error bar indicates SEM (the standard error of the mean). The *p* value was marked below the figure.
